# Intra-Operative Tissue Oxygen Tension Is Increased by Local Insufflation of Humidified-Warm CO_2_ during Open Abdominal Surgery in a Rat Model

**DOI:** 10.1371/journal.pone.0122838

**Published:** 2015-04-02

**Authors:** Jean K. Marshall, Pernilla Lindner, Noel Tait, Tracy Maddocks, Angelique Riepsamen, Jan van der Linden

**Affiliations:** 1 Illawarra Health and Medical Research Institute, University of Wollongong, Wollongong, Australia; 2 Graduate School of Medicine, University of Wollongong, Wollongong, Australia; 3 Karolinska Institute, Department of Cardiothoracic Surgery and Anesthesiology, Karolinska University Hospital, Stockholm, Sweden; 4 Moruya District Hospital, Moruya, Australia; 5 School of Women’s & Children’s Health, University of New South Wales, Sydney, Australia; Institute of Zoology, CHINA

## Abstract

**Introduction:**

Maintenance of high tissue oxygenation (PtO_2_) is recommended during surgery because PtO_2_ is highly predictive of surgical site infection and colonic anastomotic leakage. However, surgical site perfusion is often sub-optimal, creating an obstructive hurdle for traditional, systemically applied therapies to maintain or increase surgical site PtO_2._ This research tested the hypothesis that insufflation of humidified-warm CO_2_ into the abdominal cavity would increase sub-peritoneal PtO_2_ during open abdominal surgery.

**Materials and Methods:**

15 Wistar rats underwent laparotomy under general anesthesia. Three sets of randomized cross-over experiments were conducted in which the abdominal cavity was subjected to alternating exposure to 1) humidified-warm CO_2_ & ambient air; 2) humidified-warm CO_2_ & dry-cold CO_2_; and 3) dry-cold CO_2_ & ambient air. Sub-peritoneal PtO_2_ and tissue temperature were measured with a polarographic oxygen probe.

**Results:**

Upon insufflation of humidified-warm CO_2_, PtO_2_ increased by 29.8 mmHg (SD 13.3; p<0.001), or 96.6% (SD 51.9), and tissue temperature by 3.0°C (SD 1.7 p<0.001), in comparison with exposure to ambient air. Smaller, but significant, increases in PtO_2_ were seen in experiments 2 and 3. Tissue temperature decreased upon exposure to dry-cold CO_2_ compared with ambient air (-1.4°C, SD 0.5, p = 0.001).

**Conclusions:**

In a rat model, insufflation of humidified-warm CO_2_ into the abdominal cavity during open abdominal surgery causes an immediate and potentially clinically significant increase in PtO_2_. The effect is an additive result of the delivery of CO_2_ and avoidance of evaporative cooling via the delivery of the CO_2_ gas humidified at body temperature.

## Introduction

Maintenance of adequate tissue perfusion and oxygenation is a fundamental principle taught to surgeons in training [1, 2]. Low tissue oxygenation is highly predictive of surgical site infection [3–5], as tissue oxygen partial pressure (PtO_2_) drives the production of bactericidal superoxide by phagocytes [6–8]. PtO_2_ predicts collagen deposition, and therefore wound strength [9–11] and integrity or leakage of colonic anastomoses [12]. Angiogenesis depends on adequate oxygenation and is enhanced by high PtO_2_ [13]. As such, increasing peri-operative PtO_2_ is a common recommendation for the prevention of surgical complications [1, 13–16].

Interventions to increase peri-operative PtO_2_ are traditionally applied systemically, usually under the control of the anesthetist rather than the surgeon. These include manipulating inspired gases to achieve increased arterial oxygen tension (P_a_O_2_) [4, 17–19] or hypercapnia [20–23]; reduction of sympathetic vasoconstriction by minimizing pain [24], cooling [25], hypovolemia [10, 19, 26] and nicotine [18, 27]; and by the use of regional anesthesia [28–30]. However, to achieve increased PtO_2_ specifically at the surgical site, where it is required to maximize surgical site healing and to minimize surgical site sepsis, these systemic treatments all critically depend on adequate surgical site tissue perfusion and, in spite of these techniques, surgical site perfusion is frequently suboptimal [3, 10, 18]. An intervention that can locally increase surgical site perfusion and PtO_2_ may be clinically important, as it may be able to overcome poor perfusion that is often observed in surgical patients.

Insufflation of humidified-warm CO_2_ into the abdominal cavity has been proposed as a therapy to increase surgical site tissue perfusion and PtO_2_ during open abdominal surgery [31]. The CO_2_ is heated to body temperature and humidified to avoid evaporative heat loss, as evaporative cooling contributes up to 50% of heat loss during open abdominal surgery [32]. Using an active humidification system and a specially designed gas diffuser, humidified-warm CO_2_ can be diffused into the open abdominal cavity at a low velocity, while at a flow rate high enough to create a local environment with a high concentration of CO_2_ [33–36]. Both delivery of CO_2_ and maintenance of a warmer tissue temperature, by reducing evaporative heat loss, are hypothesized to have a direct vasodilatory effect on local tissue [31]. Furthermore, CO_2_ may increase PtO_2_ via the Bohr Effect, a right shift in the oxygen hemoglobin dissociation curve resulting in release of more oxygen from hemoglobin [31]. The Bohr Effect may be a direct result of both increased local CO_2_ and the subsequent decrease in local pH, and may be further amplified by reducing evaporative cooling [31].

Previous research supports the hypothesis that both exposure to CO_2_ and maintenance of tissue temperature will contribute to an increase in PtO_2_. Topical CO_2_ has been shown to increase blood flow [37–39] and PtO_2_ [39] in skin. Intra-abdominal CO_2_ has been shown to increase sub-peritoneal PtO_2_ during laparoscopic surgery compared with open abdominal surgery without CO_2_ insufflation, both in humans [40] and in a murine model [41]. Furthermore, local warming significantly increases subcutaneous PtO_2_ [25, 42] and can increase capillary blood flow by 17-fold in the presence of vasoconstriction [25]. Insufflation of humidified-warm CO_2_ into the abdominal cavity increases wound temperature during open colorectal surgery [43, 44]. However, the magnitude of the influence of insufflation of humidified or dry CO_2_ into the abdominal cavity during open abdominal surgery has not yet been measured.

This research was designed to test the hypothesis that insufflation of humidified-warm CO_2_ into the open abdominal cavity during surgery would increase local PtO_2_ due to a combination of exposure to CO_2_, as well as by reduction in evaporative cooling in the abdominal cavity. We hypothesized that insufflation of either humidified-warm CO_2_, or dry-cold CO_2_, into the abdominal cavity during open abdominal surgery would increase sub-peritoneal PtO_2_, compared with laparotomy without gas insufflation. Furthermore, we hypothesized that insufflation of humidified-warm CO_2_ would increase sub-peritoneal PtO_2_ compared with insufflation of dry-cold CO_2_.

## Materials and Methods

### Ethics and animal care

Approval for this study was granted by the University of Wollongong Animal Ethics Committee (AE 10–24). Female Wistar rats were used in strict accordance with the Australian Code for the Care and Use of Animals for Scientific Purposes [45]. All surgery was performed under isoflurane anesthesia, and all efforts were made to minimize suffering. Prior to surgery, the rats were housed two rats to a cage with ad-libitum access to food and water, and with PVC tubes as environment enrichment. The rats were maintained in a temperature controlled environment with diurnal variation of light and monitoring for signs of health every 1–2 days. Immediately following the experiments, the rats were euthanized by carbon dioxide asphyxiation, while still anesthetized.

### Induction and monitoring of general anesthesia

All rats were weighed and then pre-warmed in the animal handler’s hands for 10 minutes immediately prior to induction of general anesthesia, using a blanket and heating lamp. General anesthesia was induced with inhalant isoflurane in air via a nose cone. Throughout anesthesia the rats rested on a Small Animal Far Infra-Red Warming Pad (Kent Scientific Corporation, Connecticut, USA) to maintain body temperature. Following induction, the rats underwent endotracheal intubation with a 16 G blunted intravenous catheter under direct vision of the entrance to trachea, illuminated by a red LED shone through the skin of the throat. The endotracheal catheter was connected to a ventilator (Rodent Ventilator 7025, Ugo Basile, Varese, Italy) with ventilator gas flow of 350 ml/kg/min, with a tidal volume of 1 ml, as pilot measurements showed that this minute ventilation maintained P_a_CO_2_ in a normal range for all treatment groups. The endotracheal catheter was secured around the snout of the rat and a small piece of gauze was placed in the mouth to collect oral secretions. Depth of anesthesia was monitored via continuous measurement of heart rate and pulse pressure using a pressure transducer placed over the skin of the neck in the area of the carotid artery, and also by arterial oxygen hemoglobin saturation monitoring using a pulse oximeter (Animal Oximeter Pod ML325/AC, AD Instruments, Dunedin, New Zealand) placed on a paw. Core body temperature was measured every 5–10 minutes with a rectal thermometer (Surgipak Flexible Digital Thermometer, Vega Technologies Inc., Taipei, Taiwan). An insulating “sock” was placed over the rat’s tail to reduce radiated heat loss. A heat lamp was positioned approximately 25cm above the rat, and was only used if the rat’s temperature was falling to below 36°C. Any use of the heat lamp was recorded. Ambient temperature and humidity of the operating room was recorded with a hygrometer (HygroPalm22 Portable Humidity & Temperature Meter, Rotronic, Switzerland). Insensible fluid loss was replaced with warmed 0.9% sodium chloride delivered sub-cutaneously at 10 ml/kg/hr, once an hour, in accordance to Australian guidelines for the promotion of wellbeing of animals used for scientific purposes [46].

### Surgical procedure

Following intubation, the abdomen was clipped with an electric hair clipper (Oster Golden A5 two speed, Model 5-50A, Sunbeam Products, Florida, USA). An inverted “L” shaped laparotomy incision was then made to create adequate exposure of the parietal peritoneum for insertion of the tissue oxygen partial pressure (PtO_2_) probe. This consisted of a 60 mm long midline laparotomy incision, starting approximately 10 mm caudal to the xiphoid process. A further 40 mm long perpendicular extension of that incision was then made across the left side of the abdominal wall, from the rostral end of the first incision. A small clamp (5mm wide) was attached to the skin of the abdominal wall flap at the intersection point of the two incisions. The abdominal wall was then gently reflected towards the lower left quadrant so as to expose the parietal peritoneum. The skin was clamped so as to minimize tension on the peritoneum. To further expose the parietal peritoneum, by way of relaxation of the left abdominal wall, the left hind leg was flexed and secured using tape across the foot.

### Measurement of Tissue Oxygen Partial Pressure

PtO_2_ was measured using a combined temperature and polarographic oxygen tension probe (Licox CC1P1, Gesellschaft fu¨r Medizinische Sondensysteme, GmBH, Kiel, Germany). Each probe is calibrated by the manufacturer and is supplied with an individual calibration card that is inserted into the monitor prior to use. The accuracy of the probe is ±10% for PtO_2_ and ± 0.2 C for temperature. To position the PtO_2_ probe a 16G intra venous catheter was tunneled beneath the peritoneal membrane from the lower left quadrant to the upper left quadrant under direct vision. The needle was then removed and the PtO_2_ probe was inserted into the catheter. Finally, the catheter was gently retracted so that a minimum of 30 mm length of probe remained within the tissue, ensuring that the measurement portion of the probe was fully embedded. Dissection conducted during pilot investigations showed that the probe was embedded in the musculature of the abdominal wall. The PtO_2_ probe was then connected to a Licox CMP Oxygen and Temperature Monitor (Gesellschaft fu¨r Medizinische Sondensysteme, GmBH, Kiel, Germany) to allow continuous recording of PtO_2_ and tissue temperature throughout the experiment.

### Experimental design

Three sets of experiments were conducted in a randomized cross-over design. The first treatment was randomized and then treatment was alternated so that each rat received both treatments at least twice. Treatments were administered for a minimum of 15 minutes, ensuring that any initial steep change in PtO_2_ was captured in its entirety.

Experiment 1: 5 rats alternatively exposed to insufflation of humidified-warm CO_2_ or ambient air

Experiment 2: 7 rats alternatively exposed to insufflation of humidified-warm CO_2_ or dry-cold CO_2_ insufflation

Experiment 3: 3 rats alternatively exposed to insufflation of dry-cold CO_2_ or ambient air

Sample size calculation for experiment one was based on a minimal clinically significant difference between conditions of 15 mmHg, between subject standard deviation of 35 mmHg, correlation between observation on the same subject as 0.7, α = 0.0, β = 0.20, and assuming two crossovers per animal. Sample size calculations for the subsequent experiments were carried out at the completion of each experiment with the aim of minimizing the number of animals used. This resulted in a different number of animals per experiment.

### Treatment conditions

Preliminary testing showed that the abdominal cavity of a rat is too shallow to create a local environment of high CO_2_ gas concentration. This is due to the relatively thin abdominal wall of the rat compared with humans, and the minimal use of retraction in this model in order to protect the peritoneum from physical trauma. To ensure the abdomen was exposed to a stable high concentration of CO_2_, the rat was placed in a plastic container with a 9 x 12 cm hole in the top through which the CO_2_ was insufflated. CO_2_ being heavier than air, air was then displaced from the container by the CO_2_ and the abdomen was in a stable, high CO_2_ concentration environment. The CO_2_ was continuously insufflated into the container at 9 L/min via a gas diffuser (VitaDiffuser, Cardia Innovation, Sweden) that ensures the gas enters the container at a low velocity, thereby reducing turbulence whilst allowing high CO_2_ gas concentrations within the container. The CO_2_ entered the container at the diffuser and continually overflowed out of the hole at the top of the container, creating a continuous flow of CO_2_ over the abdominal wound. Pilot measurements of CO_2_ concentration using a CheckMate II gas analyzer (PBI Dansensor, Denmark) showed that the environment within the box is maintained at > 90% CO_2_. The ability to create an environment of high CO_2_ concentration within a surgical cavity has been well documented, both with and without humidification [33, 35, 36]. In the humidified-warm CO_2_ conditions, the CO_2_ was humidified and warmed using a humidifier controller and delivered to the gas diffuser by a heated delivery tube (HumiGard, Fisher and Paykel Healthcare, New Zealand). Independent testing has shown that the humidifier delivers >98.0% relative humidity at 37°C [47].

### Data analysis

PtO_2_ and tissue temperature measurements were averaged over the last minute for each treatment condition. Each treatment was paired with the alternate treatment (the first treatment with the second, third with the fourth etc.) to give a change in PtO_2_ and tissue temperature for each cross-over trial. The Shapiro-Wilk test was used to check the normality of the data set for each experiment. When the assumption of normality was satisfied, a paired Student’s t-test was conducted to test whether the mean change between paired treatment conditions differed from zero. When the assumption of normality was not satisfied, a Hodges-Lehman median difference and Wilcoxon Signed Rank test was used. P-value <0.05 was considered statistically significant for all tests.

## Results

### Animals and arterial oxygen saturation

The average weight of the rats was 294 g (SD 27), and did not differ between experiments (p = 0.34). There was no difference in arterial oxygen saturation (SpO_2_) during PtO_2_ data collection between conditions in any of the experiments (p = 0.48, 0.51, 0.46 for experiments 1–3)

### Sub-peritoneal tissue oxygenation partial pressure (PtO_2_) and tissue temperature

**Fig 1:**
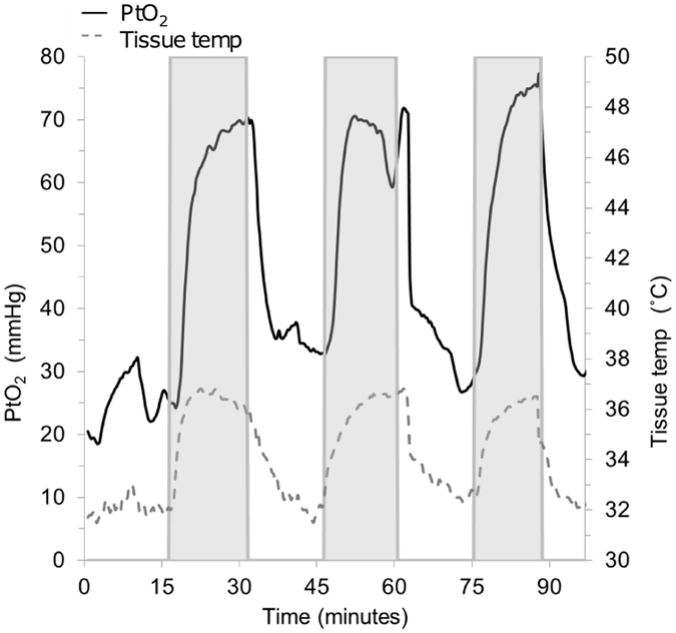
Representative data from one rat in experiment 1 (Insufflation of humidified-warm CO_2_ vs exposure to ambient air). The shaded areas show each period of insufflation of humidified-warm CO_2_. A rapid increase in both PtO_2_ and tissue temperature is seen each time gas insufflation is started, and is reversed when insufflation is stopped.

**Fig 2:**
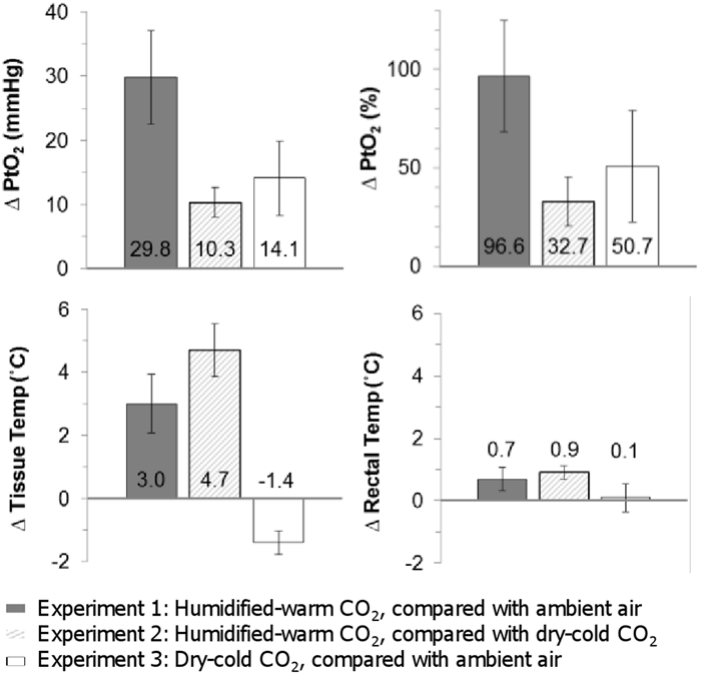
Change in PtO_2_ (shown as both an absolute change (mmHg) and relative change (%)), tissue temperature and rectal temperature affected by the intervention condition of each experiment. Error bars show 95% confidence intervals. Where error bars do not cross zero, the intervention had a statistically significant effect compared with the control condition.

Upon insufflation of humidified-warm CO_2_, both PtO_2_ and tissue temperature increased almost immediately following the start of each period of gas insufflation, and was reversed each time gas insufflation was stopped (Fig. 1). Mean sub-peritoneal PtO_2_ increased by 29.8 mmHg (SD 13.3, p<0.001), or 96.6% (SD 51.9), and tissue temperature by 3.0°C (SD 1.7 p<0.001) compared with exposure to ambient air (experiment 1) (Table 1 and Fig. 2).

**Table 1:** Tissue oxygen tension (PtO_2_) and tissue temperature results for each experiment.

Experiment	Intervention	Control	Number of rats	Number of trials	PtO_2_			Tissue Temperature				
					Mean Control (mmHg)	Mean change with intervention (mmHg)	p	Mean Control (°C)	Mean change with intervention (°C)	p
						Compared to control			Compared to control	
1	Humidified—warm CO_2_	Ambient air	5	13	33.2 (8.2)	29.8 (13.3)	<0.001	34.0 (1.6)	3.0 (1.7)	<0.001
2	Humidified—warm CO_2_	Dry—cold CO_2_	7	20	33.3 (7.2)	10.3 (5.1)	<0.001*	31.4 (1.6)	4.7 (1.9)	<0.001
3	Dry—cold CO_2_	Ambient air	3	7	28.0 (7.2)	14.1 (7.2)	0.005	32.1 (1.4)	-1.4 (0.5)	0.001

All results show ‘mean (standard deviation)’ unless stated

* Related-samples Hodges-Lehman median difference shown, and Wilcoxon matched pair signed rank test used as assumption of normality was not satisfied.

Smaller, but significant, increases in PtO_2_ were seen when the two components of the therapy, exposure to humidity/warmth and exposure to CO_2_, were observed separately. Experiment 2 showed an increase in PtO_2_ of 10.3 mmHg (SD 5.1 p<0.001), or 32.7% (SD 16.6) upon the delivery of humidity and warmth in the constant presence of CO_2_ insufflation. Experiment 3 showed an increase in PtO_2_ of 14.1 (SD 7.2, p = 0.005), or 50.7% (SD 37.9), upon insufflation of dry-cold CO_2_ compared with exposure to ambient air.

With a similar pattern to PtO_2_, tissue temperature increased upon the addition of humidity and warmth by 4.7°C (SD 1.9, p < 0.001), in the constant presence of CO_2_ insufflation (experiment 2). However, in contrast to PtO_2_, tissue temperature decreased by 1.4°C (SD 0.5, p = 0.001) during insufflation of dry-cold CO_2_ compared with exposure to ambient air (experiment 3).

### Body temperature maintenance

There was an increase in body temperature following insufflation of humidified-warm CO_2_ of 0.7°C (SD 0.7, p = 0.001) when compared to exposure to ambient air (experiment 1), and 0.9°C (SD 0.5, p < 0.001) when compared to exposure to dry-cold CO_2_ (experiment 2) (Fig. 2). There was no significant change in body temperature during insufflation of dry-cold CO_2_ compared to exposure to ambient air (experiment 3) (Fig. 2). There was no significant difference in the average heat pad setting between conditions in any of the experiments. The heat lamp was normally not required to maintain normothermia, but was used half as often in the conditions in which humidity and warmth were delivered.

## Discussion

This research was designed to test the hypothesis that insufflation of humidified-warm CO_2_ into the open abdominal cavity during surgery would increase local PtO_2_ by a combination of the delivery of both CO_2_ and by reducing evaporative cooling in the peritoneal cavity. The first set of randomized cross-over trials showed that insufflation of humidified-warm CO_2_ causes an immediate and significant increase in PtO_2_ compared with exposure to ambient air, an average increase of 29.8 mmHg or 96.6%. Two subsequent sets of randomized cross-over trials showed that exposure to CO_2_ and reduction of evaporative cooling by exposure to humidity/warmth separately have an additive effect on PtO_2_. Furthermore, the increase in PtO_2_ upon the delivery of humidity/warmth may be explained by a concomitant increase in tissue temperature, in contrast to the drop in tissue temperature observed when dry-cold CO_2_ was insufflated in comparison with ambient air. To our knowledge this is the first report of the effect of CO_2_ insufflation during open abdominal surgery on PtO_2_.

The observed increase in PtO_2_ upon exposure to CO_2_ is consistent with previous reports of increased PtO_2_ upon topical CO_2_ application to the skin [39], and intra-abdominal exposure of CO_2_ during laparoscopy [40, 41]. The enhanced maintenance of tissue and core body temperature observed upon exposure to humidified-warm CO_2_ is also consistent with previous reports in human trials in colo-rectal surgery [43, 44]. Furthermore, the decrease in local tissue temperature observed with exposure to dry-cold CO_2_ is also consistent with human results that showed decreased local tissue temperature with the use of dry-cold CO_2_ in open cardiac surgery [48].

The 30 mmHg increase in PtO_2_ observed upon insufflation of humidified-warm CO_2_ is higher than the threshold of 15–25 mmHg that is widely considered as clinically significant [3, 49, 50]. A 25 mmHg increase in PtO_2_ has been shown to predict a 30% drop in surgical site infection rate [3]. Furthermore, a 40–50% fall in PtO_2_ is highly predictive of anastomotic leakage [12], suggesting that the 97% increase in PtO_2_ measured upon insufflation of humidified-warm CO_2_ may have a clinically important positive impact of anastomotic healing. Importantly, the results suggest that this local (surgical field) therapy may be at least as effective at increasing PtO_2_ during the operative period as systemically applied intraoperative therapies. An increase in intra-operative P_a_O_2_ from 150 to 300 mmHg increases PtO_2_ by approximately 19–20 mmHg in non-obese patients [17]. The increase in PtO_2_ drops to 11 mmHg in obese patients, likely due to reduced perfusion related to adiposity [17]. Manipulation of end tidal CO_2_ from 30 to 45 mmHg increases PtO_2_ by 12–16 mmHg [20, 23]. The observed effect of humidified-warm CO_2_ on PtO_2_ in the current study is larger than that achieved by aggressive fluid management [26], and by the use of thoracic epidural anesthesia [28–30].

While intra-abdominal insufflation of humidified-warm CO_2_ during open abdominal surgery may cause an increase in PtO_2_ of a similar magnitude to systemic therapies, the innovation of intra-abdominal insufflation of humidified-warm CO_2_ is that it achieves a local increase in PtO_2_ that may be able to overcome limitations of systemic therapies. The results of the current study suggest that a local effect was achieved, as the change in tissue temperature in the surgical site (sub-peritoneal in the abdominal wall) was at least 4-fold greater than the observed change in rectal temperature. It is likely that the insufflation of humidified-warm CO_2_ locally increases tissue oxygenation by mimicking normal metabolic regulation of oxygen delivery. This is most likely a combination of both a local increase in micro-perfusion through vasodilation, and a decrease in oxygen hemoglobin affinity. Increase in perfusion has previously been measured in response to topical CO_2_ [37–39] and local heating [25], and has been reported with concomitant increase in PtO_2_ [25, 39]. Delivery of oxygen to wounds is critically dependent on micro-perfusion to the surgical site. Oxygen dissipates radially from the vasculature to the tissue and PtO_2_ can drop rapidly just 20 μm from the vessel wall [51]. Micro-perfusion is dramatically altered by vasoconstriction and by arteriovenous shunts, which can allow oxygenated blood to bypass capillary beds resulting in higher venous PO_2_ than capillary PO_2_ [51]. The potential for inadequate perfusion to the surgical site is an obstructive hurdle for the effectiveness of systemic interventions to increase PtO_2_ [52]. We propose that maintenance of a warm, humid, high CO_2_ surgical site environment during open surgery in the abdomen is a solution that appears to increase perfusion directly at the target site and thereby increase local tissue oxygenation.

The implementation of intra-abdominal humidified-warm CO_2_ in open abdominal surgery into clinical practice is simplified by the fact that intra-abdominal CO_2_ is already established as the recommended gas for the creation of pneumoperitoneum for laparoscopic surgery [53]. Due to early concerns, the effect of intra-abdominal CO_2_ exposure has been broadly investigated, especially in respect to post-operative adhesion formation and tumor implantation. Recent mechanistic animal research concludes that exposure to dry CO_2_ pneumoperitoneum does not increase post-operative adhesion formation compared to exposure to dry air, when pneumoperitoneum is established at an appropriately low pressure and with adequate ventilatory support so as to avoid tissue hypoxia [41, 54, 55]. Furthermore, human trials have found that the rates of adhesive bowel obstruction [56, 57] and cancer survival [58] for laparoscopic surgery with CO_2_ pneumoperitoneum are at least on-par with open abdominal surgery. In light of this evidence, a recent study that suggested a CO_2_ operating environment increases adhesion formation [59] has been criticized as not using an adequately standardized adhesion model [60]. Another point of clinical interest is the fate of the excess CO_2_ that overflows from an open abdominal cavity. The Coanda effect causes the CO_2_ to flow out of the wound attached to the adjacent surface, similar to gas flow in avionics. A combination of the negative buoyancy of CO_2_ and the Coanda effect causes the CO_2_ to drop to the floor of the operating room and therefore operating staff are not exposed to high inspired CO_2_ [61, 62].

The rat model used for this research included endotracheal intubation, replacement of insensible fluid loss, and continuous monitoring of depth of anesthesia by a dedicated anesthetist. Although applicability to the human clinical setting is inferred, there is some evidence that the PtO_2_ response is similar between species, as similar increases in PtO_2_ have been reported in rodent [41] and human [4]surgical models in response to increased inspired oxygen. The use of a rat model had several advantages over measurement in humans. Most notably, the model allowed for the expensive PtO_2_ probe to be re-used for several rats, avoiding the expense of single use probes that would be required in the human clinical setting. This enabled several different experiments to be conducted, which would not have been economically feasible in a human model. The model also allowed complete assurance that the PtO_2_ probe remained in place and that the overlaying tissue remained free of surgical fluids, blood or bowel.

Continuous monitoring of arterial CO_2_ partial pressure (P_a_CO_2_) was not conducted in this model, as we determined that the extra complication of femoral artery cannulation in an already long procedure was not justified. It is possible that a systemic increase in P_a_CO_2_ contributed to the measured increase in PtO_2_. During the development of the current rat model, blood gas measurements were taken at different minute ventilation rates in 6 rats with and without humidified-warm CO_2_ insufflation. The results, supplied in supplemental files, suggest that insufflation of humidified-warm CO_2_ increases P_a_CO_2_ by 7 mmHg at the minute ventilation used in the current study (note that the results were not statistically significant and therefore did not justify increasing minute ventilation during CO_2_ insufflation in this protocol). Human data suggests that a 7 mmHg increase in P_a_CO_2_ would increase PtO_2_ by just 5–7 mmHg [20, 23]. It is, therefore, unlikely that an increase in P_a_CO_2_ alone is sufficient to explain the large increase in PtO_2_ observed in the current study.

In conclusion, the current study has shown that insufflation of humidified-warm CO_2_ into the abdominal cavity during open abdominal surgery in a rat model causes an immediate and clinically significant increase in PtO_2_. Furthermore, the effect is an additive result of the delivery of CO_2_ and avoidance of evaporative cooling via the delivery of the gas humidified at body temperature. This finding may have important clinically implications, as the local application of the therapy may be able to overcome inadequate local micro-perfusion, which limits delivery of supplemental oxygen to the surgical site in many surgical patients.

## Supporting Information

S1 Dataset This contains the raw data results for experiment 1. (XLSX)

S2 Dataset This contains the raw data results for experiment 2. (XLSX)

S3 Dataset This contains the raw data results for experiment 3. (XLS)

S4 Dataset This contains blood gas data collected from the experimental model and different minute ventilation rates with and without humidified-warm CO_2_ insufflation. (XLS)
